# Amifostine Pretreatment Attenuates Myocardial Ischemia/Reperfusion Injury by Inhibiting Apoptosis and Oxidative Stress

**DOI:** 10.1155/2017/4130824

**Published:** 2017-03-14

**Authors:** Shao-ze Wu, Lu-yuan Tao, Jiao-ni Wang, Zhi-qiang Xu, Jie Wang, Yang-jing Xue, Kai-yu Huang, Jia-feng Lin, Lei Li, Kang-ting Ji

**Affiliations:** Department of Cardiology, The Second Affiliated Hospital and Yuying Children's Hospital, Wenzhou Medical University, Wenzhou 325000, China

## Abstract

The present study was aimed at investigating the effect of amifostine on myocardial ischemia/reperfusion (I/R) injury of mice and H9c2 cells cultured with TBHP (tert-butyl hydroperoxide). The results showed that pretreatment with amifostine significantly attenuated cell apoptosis and death, accompanied by decreased reactive oxygen species (ROS) production and lower mitochondrial potential (ΔΨm). In vivo, amifostine pretreatment alleviated I/R injury and decreased myocardial apoptosis and infarct area, which was paralleled by increased superoxide dismutase (SOD) and reduced malondialdehyde (MDA) in myocardial tissues, increased Bcl2 expression, decreased Bax expression, lower cleaved caspase-3 level, fewer TUNEL positive cells, and fewer DHE-positive cells in heart. Our results indicate that amifostine pretreatment has a protective effect against myocardial I/R injury via scavenging ROS.

## 1. Introduction

Ischemia state for a long time can cause irreversible myocardial necrosis. What is more, the condition does not improve but worsen after myocardial reperfusion, causing myocardial ultrastructure, metabolism, and electrophysiological damage, known as ischemia-reperfusion injury [1–3].

In the past decades, with the introduction of thrombolytic therapy [4], arterial bypass surgery [5], percutaneous transluminal coronary angioplasty [6], and cardiopulmonary bypass, [7] patient survival after heart attack has been significantly improved. However because of ischemia-reperfusion injury, the prognosis of patients is still grim. The underlying mechanisms of ischemia-reperfusion injury include free radical damage, calcium overload, leukocyte activation, and microvascular damage [8–10]. Myocardial ischemia-reperfusion injury will produce a large number of free radicals, leading oxidation, crosslinking, denaturation, and degradation of DNA, RNA, protein, and polysaccharide molecules and eventually causing the myocardial cell apoptosis and death [11]. Therefore, in the process of myocardial ischemia reperfusion, increased production of free radicals should be halted.

Amifostine is the most effective one among more than 4000 kinds of radiation protective agents which were compounded by the United States Institute of Walter Reed Army during the cold war. It is also the first American FDA approved generic cell protective agent. Studies indicated that amifostine can be selectively absorbed by normal cells, with low concentration in tumor cells [12, 13]. Alkaline phosphatase in the cellular membrane of endothelial cells of small vessels induces the dephosphorylation of amifostine into the active metabolite free thiol WR-1065, which acts as a free radical scavenger and protect normal cells in the radiotherapy [14].

The aim of our study was to determine if amifostine could prevent against myocardial ischemia-reperfusion injury and explore the underlying mechanisms.

## 2. Materials and Methods

### 2.1. Animals and H9c2 Cells Culture

Male C57BL/6 mice (20–25 g) aged 6-7 weeks were purchased from the SLAC Laboratory Animal Center of Shanghai. Mice were housed in a SPF animal room of Wenzhou Medical University Animal Center, fed with a chow diet and water available ad libitum under a constant room temperature with a 12 : 12 h light-dark cycle. H9c2 were purchased from American Type Culture Collection (ATCC, Manassas, VA, USA). H9c2 cells were cultured in high glucose DMEM with 10% (v/v) fetal bovine serum (FBS) without penicillin/streptomycin in a humidified atmosphere (5% CO_2_, 21% O_2_) at 37°C. The medium was refreshed every 2 days. A stock of H9c2 cells were grown in a 100 mm Petri dish [15].

### 2.2. Ischemia and Reperfusion Model

All animal experiments followed the Wenzhou Medical University Policy on the Care and Use of Laboratory Animals. All experimental procedures were approved by the Committee on the Ethics of Animal Experiments of Wenzhou Medical University (Number: wydw2014-0058). The myocardial I/R injury in C57BL/6 mice was induced as described previously [16, 17]. Briefly, the mice were anesthetized by inhaling isoflurane. Then the heart was exposed by a lateral cut along the up-margin of the third or fourth rib. After the exposure of the entire left anterior descending coronary artery (LAD) system, a ligation was performed using a 7-0 silk suture at the distal 1/3 of the LAD. The ligation was released after coronary artery occlusion for 30 min and reperfusion for 4 h. Animals in the Sham group were also anesthetized, and a suture was passed under the LAD without occlusion.

### 2.3. Treatments

The mice were randomly divided into four groups: (1) the Sham group: mice were anesthetized and a suture was passed under the LAD without occlusion; (2) the I/R control group: mice were pretreated with 0.9% normal saline (NS); (3) the I/R + L group: mice were pretreated with amifostine (80 mg/kg i.v.) in 0.2 ml 0.9% NS; (4) the I/R + H group: mice were pretreated with amifostine (400 mg/kg i.v.) in 0.2 ml 0.9% NS. Amifostine was given 30 min before ischemia induction. The coronary occlusion lasted 30 min followed by reperfusion for 4 h. At the end of reperfusion, blood was collected from common carotid artery and the heart was harvested.

### 2.4. Myocardial Infarct Size Measurement

The myocardial infarct size was assessed by Evan's Blue and triphenyltetrazolium chloride (TTC) double staining method [18]. At the end of reperfusion, the LAD was reoccluded and 0.2 ml of a 2% Evan's Blue dye was injected into the inferior vena cava. When the right side of the heart turned blue, the heart was quickly removed, rinsed in NS, and frozen at −20°C. Five 1 mm thick heart sections were made and incubated in 1% TTC for 20 min at 37°C. The viable tissue which was stained red by TTC was defined as area at risk (AAR). The nonischemic myocardium was stained deep blue by Evans Blue. Infarct area (INF) appeared pale after staining. A percent of infarcted area over total area at risk (% INF/AAR) was calculated.

### 2.5. Myocardial TUNEL Staining and Dihydroethidium Staining

After 4 hours' reperfusion, the heart was removed. Formalin-fixed heart tissues were then embedded in paraffin and approximately 5 *μ*m thick sections were cut. TUNEL staining was used to detect cardiomyocyte apoptosis. The section images were taken by a confocal microscopy (NIKON A1R/A1, Nikon, Japan). For dihydroethidium (DHE) staining, fresh frozen sections were incubated with 10 *μ*mol/L of DHE (Beyotime Biotechnology, Shanghai) at 37°C for 30 minutes, and then DAPI was applied. Images were acquired by fluorescence microscope.

### 2.6. Determination of Superoxide Dismutase (SOD) Activity and Malondialdehyde (MDA) Levels

The SOD activity and MDA levels were measured on frozen myocardial tissue, which was conducted using commercial kit reagents (JianCheng Bioengineering Institute, Nanjing, China) according to the manufacturers' instructions.

### 2.7. Cell Viability

H9c2 cells were seeded into 96-well plates at a concentration of 5000 cells per well. The cells were pretreated with amifostine (0.78125, 1.5625, 3.125, 6.25, 12.5, 25, 50, and 100 *μ*M) for 30 min before being exposure to tert-Butyl hydroperoxide (TBHP) for 12 h. The number of viable cells was evaluated by MTT assay. Briefly, MTT dye solution was added to each well and incubated for 4 h. The number of viable cells was measured by evaluating Absorbance at 490 nm. The MTT assay was repeated three times for consistency.

### 2.8. Measurement of Reactive Oxygen Species (ROS) and Mitochondrial Membrane Potential (ΔΨ*m*)

We used TBHP, a more stable chemical than H_2_O_2_, to induce oxidative stress. For measurement of ROS of the H9c2 cells, cells were incubated with 10 *μ*mol/L ROS sensitive dye 2′,7′-dichloruoresceindiacetate (DCFH-DA) at 37°C for 20 min. ROS was detected by a flow cytometry sorter (BD Biosciences, San Jose, CA, USA) and quantified by BD FACS software. The above experiments were repeated three times. ΔΨ*m* was measured using JC-1 staining; cells were seeded into Petri dishes. After treatment, the dishes were incubated in JC-1 staining solution (5 mg/ml) at 37°C for 20 min. Subsequently the staining cells were washed twice with JC-1 staining buffer; images were taken by a confocal laser scanning microscopy.

### 2.9. Determination of Apoptosis Ratio

H9c2 cells (1.3 × 10^5^) were seeded in six-well plates for 24 h. Following different treatments, cells of each group were collected, washed with phosphate-buffered saline (PBS) twice, and resuspended at a density of 1 × 10^6^/ml. Next, cells (500 *μ*l) were incubated with 5 *μ*l annexin V-fluorescein isothiocyanate and 10 *μ*l propidium iodide (PI, 20 *μ*g/mL) for 15 min in the dark at room temperature. The fluorescence intensities of annexin V/PI-stained cells were analyzed by a flow cytometry within 1 h. The apoptotic cells, including annexin V^+^/PI^−^, were counted. The apoptosis ratio was quantified by BD FACS software. The above experiments were repeated six times.

### 2.10. Western Blot Analysis

Protein was prepared from homogenized myocardial tissue samples. Protein concentration was measured with BCA assay. Western blot analysis was performed as previously described [19]. The following primary antibodies were used: anticleaved caspase-3 (1 : 1000, Bioworld), anti-Bcl-2 (1 : 500, Abcam), anti-Bax (1 : 1000, Abcam), anti-SOD1 (1 : 2000, Abcam), anti-SOD2 (1 : 5000, Abcam), and anti-glyceraldehyde 3-phosphate dehydrogenase (GAPDH, 1 : 1000, Abcam). In next day immunoreactive bands were detected by incubating with anti-mouse or anti-rabbit secondary antibody conjugated with horseradish peroxidase (1 : 500, Abcam) and visualized using enhanced chemiluminescence reagents (Bio-Rad, Hercules, CA). The amounts of the proteins were analyzed using Image Pro Plus and normalized to their respective controls.

### 2.11. Statistical Analysis

All the date are presented as the mean ± standard error of the mean (SEM). The differences of all measured parameters were assessed by One-way ANOVA followed by post hoc tests. Data were analyzed using GraphPad Prism-5 statistic software (GraphPad software, Inc., La Jolla, CA). A difference of *P* < 0.05 was considered statistically significant.

## 3. Results

### 3.1. Effect of Amifostine on Myocardial Infract Size

**Figure 1:**
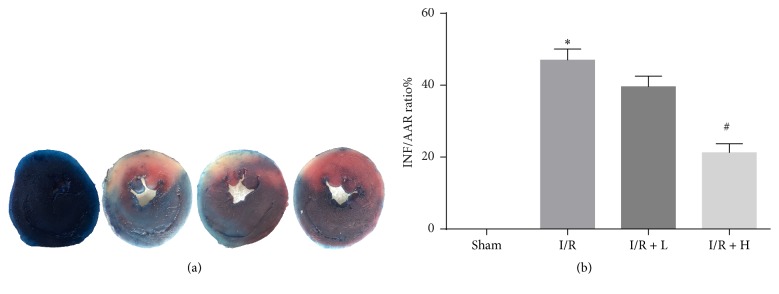
Amifostine pretreatment reduces myocardial I/R injury. (a) Representative images of the infract area (INF: white), area at risk (AAR: red and white), and normal area (blue). (b) Quantitative analysis of infarct size and the ratio of INF/AAR. Data are shown as means ± SEM; ^*∗*^*P* < 0.05, I/R (pretreated with NS) control group versus Sham group; ^#^*P* < 0.05 I/R + H (pretreated with amifostine 400 mg/kg) group versus I/R control group; *n* = 6 per group.

To evaluate the protective effect of amifostine on myocardial I/R injury, the myocardial infarct size was measured. The effects of amifostine treatment on the infarct size and necrosis in mice are shown in Figure 1. Myocardial infarct area was indicated in pale color in the I/R control group. Compared with the I/R control group, the I/R + H group had a significant improvement in INF/AAR ratio suggesting that pretreatment with amifostine can reduce myocardial infarct area.

### 3.2. Effect of Amifostine Pretreatment on H9c2 Cells Viability

**Figure 2:**
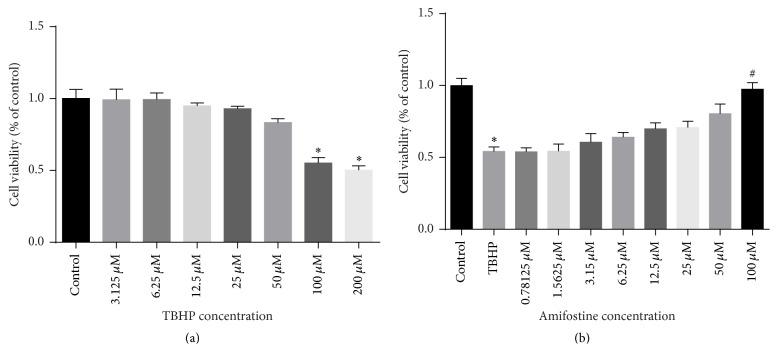
Effect of amifostine pretreatment on H9c2 cell viability. (a) Effects of different concentrations of TBHP on H9c2 cell viability. The cell viability was markedly inhibited in the 100 *μ*M and 200 *μ*M TBHP groups. (b) Effects of amifostine pretreatment on H9c2 cell viability subjected to TBHP. Pretreatment with 100 *μ*M amifostine group significantly enhanced cell viability compared with the TBHP group. Data are shown as means ± SEM; ^*∗*^*P* < 0.05, 100 *μ*M TBHP group or 200 *μ*M TBHP group versus control group; ^#^*P* < 0.05, 100 *μ*M amifostine versus TBHP group; *n* = 6 per group.

To evaluate the protective effect of amifostine on H9c2 cells, H9c2 cells were cultured with different concentrations of TBHP to mimic an I/R injury (Figure 2(a)). Cell viability of 100 *μ*M TBHP group and 200 *μ*M TBHP group was significantly reduced compared with the control group. So, in the subsequent cell experiments 100 *μ*M TBHP was used to induce cell injury. Pretreatment with amifostine alleviated TBHP-induced cell injury in a dose-dependent manner (Figure 2(b)). The subsequent cell experiments were performed using amifostine at a final concentrations of either 100 *μ*M (high dose group) or 25 *μ*M (low dose group).

### 3.3. Amifostine Attenuated TBHP-Induced H9c2 Cell Apoptosis

**Figure 3:**
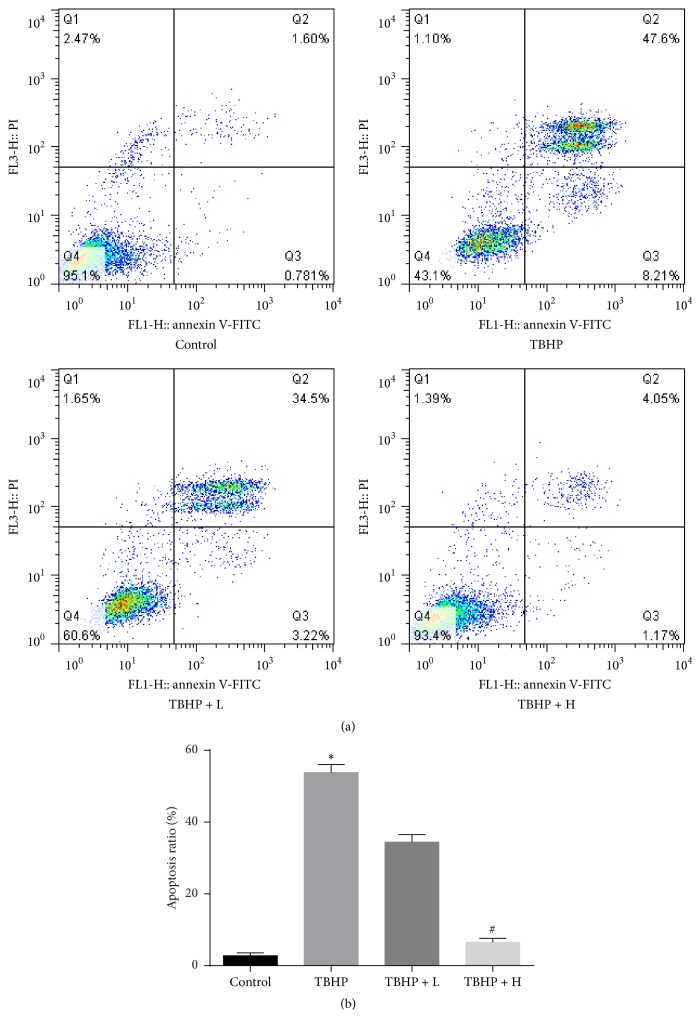
Effect of amifostine pretreatment on TBHP-induced apoptosis in H9c2 cells. (a) Typical images of flow cytometry. (b) The apoptosis ratio was quantified by BD FACS software. Amifostine pretreatment (TBHP + H group) significantly reduced apoptosis compared with the TBHP group. Data are shown as means ± SEM; ^*∗*^*P* < 0.05, TBHP group versus control group, ^#^*P* < 0.05, TBHP + H group versus TBHP group; *n* = 6 per group.

Apoptosis is an important index of myocardial I/R injury. We explored if amifostine (25 *μ*M and 100 *μ*M) pretreatment could prevent TBHP-induced H9c2 cell apoptosis (Figure 3). Data showed apoptosis could be significantly reduced with pretreatment of 100 *μ*M amifostine when cells were exposed to 100 *μ*M TBHP for 12 h, indicating that amifostine could exert cardioprotective effects by inhibiting apoptosis.

### 3.4. Effect of Amifostine on Mitochondrial Membrane Potential (ΔΨ*m*)

**Figure 4:**
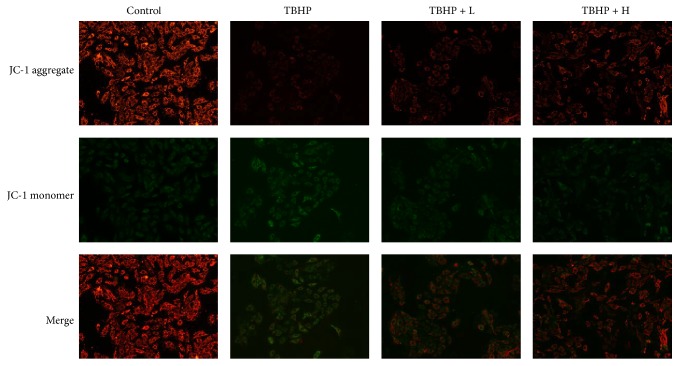
The effect of amifostine pretreatment on mitochondrial membranes potential changes (ΔΨ*m*) in H9c2 cells cultured with TBHP. The ΔΨ*m* of TBHP group remarkably decreased compared with control group; the ΔΨ*m* in TBHP + H group was increased compared to the TBHP group. *n* = 6 per group.

Mitochondrial membrane potential (ΔΨ*m*) will decrease when apoptosis happens. In order to further confirm the antiapoptosis effect of amifostine, H9c2 cells were pretreated with 25 *μ*M and 100 *μ*M amifostine, respectively, before exposure to TBHP (100 *μ*M, 12 h) (Figure 4). Depolarization of ΔΨ*m* was then measured by confocal microscope using JC-1 probe. Red and green fluorescence images represented polarized and depolarized mitochondria, respectively, while the ratio of red to green represented the depolarization ratio of ΔΨ*m*. Date showed that ΔΨ*m* of the TBHP group decreased remarkably compared with the control group, and this was prevented in the TBHP + H group.

### 3.5. Myocardial TUNEL Staining

**Figure 5:**
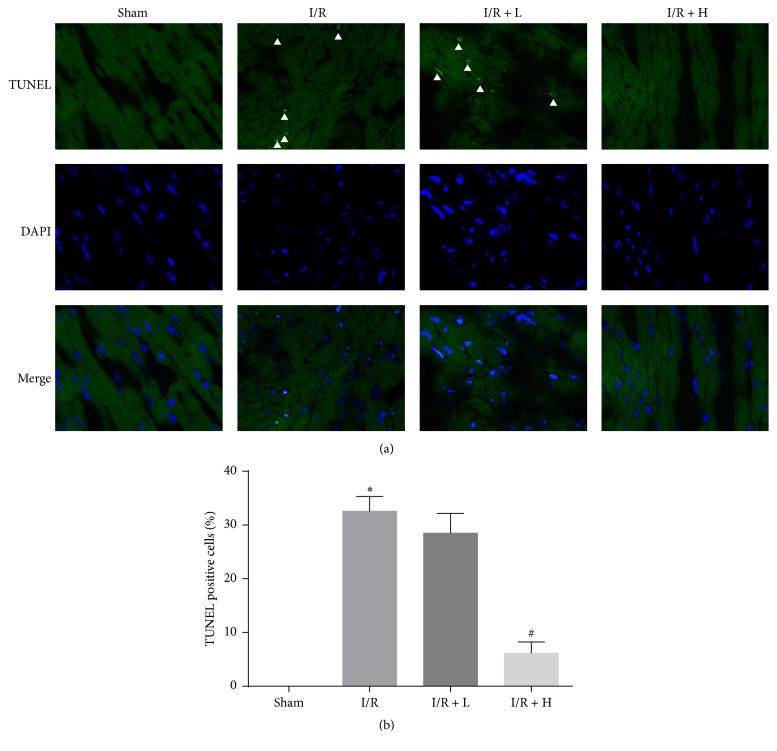
The effect of amifostine pretreatment on myocardial TUNEL staining. (a) Representative confocal microscopy images. White triangle indicates positive staining cells (green). DAPI staining (blue) indicates nucleus. Magnification: ×400. (b) Percentages of TUNEL positive cells of total cells. Data are shown as means ± SEM; ^*∗*^*P* < 0.05, I/R group versus Sham group, ^#^*P* < 0.05, I/R + H group versus I/R group; *n* = 6 per group.

In the mouse myocardial I/R model, we used TUNEL staining to evaluate apoptosis. I/R induced significant myocardial apoptosis as expected. However, in the I/R + H group, apoptosis was attenuated (Figure 5).

### 3.6. Myocardial DHE Staining

**Figure 6:**
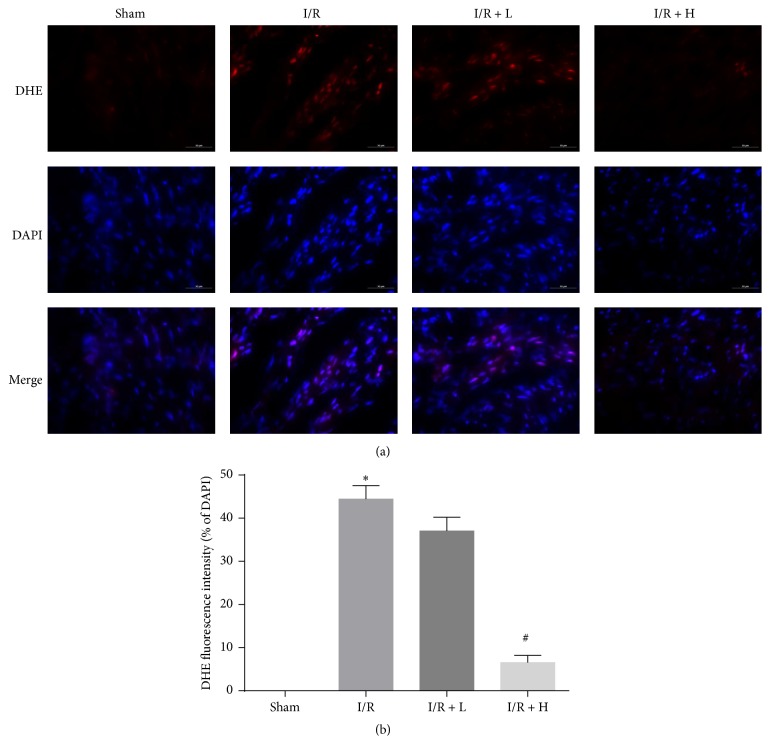
The effect of amifostine pretreatment on myocardial DHE staining. (a) Representative fluorescent microscopy images with positive staining cells (red). DAPI staining (blue) indicates nucleus. Magnification: ×400. (b) Percentages of DHE-positive cells of total cells. Data are shown as means ± SEM; ^*∗*^*P* < 0.05, I/R group versus Sham group, ^#^*P* < 0.05, I/R + H group versus I/R group; *n* = 6 per group.

To investigate the antioxidant activities of amifostine, heart tissues were stained with DHE as a fluorescent marker of ROS. Many DHE-positive cells were observed in the I/R group, indicating that I/R induced generation of ROS. High dose amifostine effectively reduced the generation of ROS caused by I/R (Figure 6).

### 3.7. Effect of Amifostine on the Expression Levels of Antioxidant and Apoptosis Related Proteins

**Figure 7:**
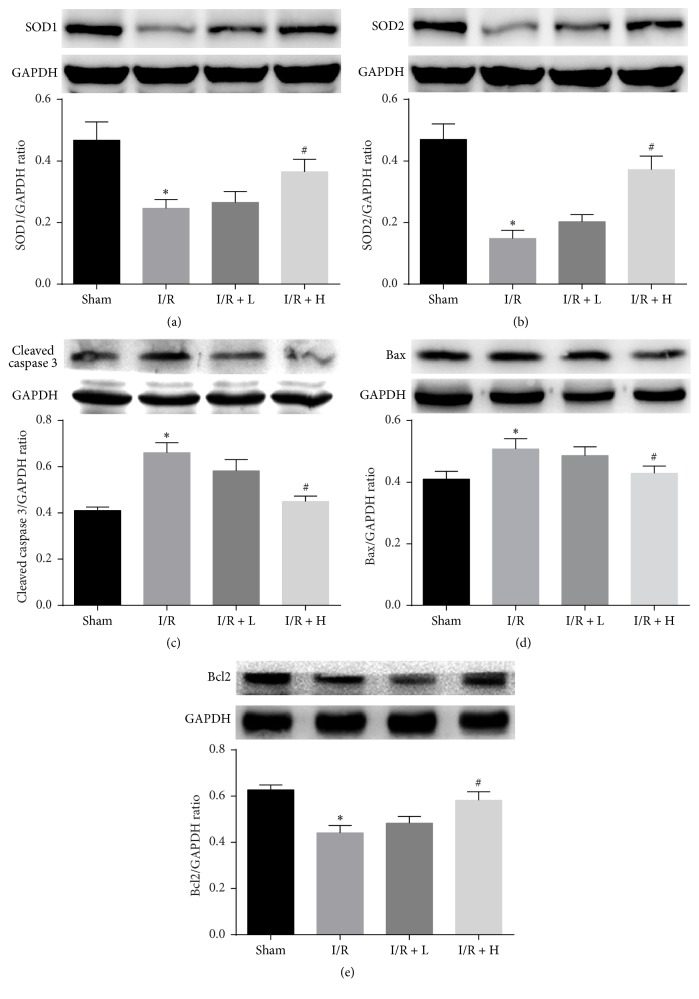
Effects of amifostine pretreatment on SOD1, SOD2, cleaved caspase-3, Bax, and Bcl-2 expressions in myocardial tissues. (a) Representative bands of cleaved SOD1 and quantitative analysis results. (b) Representative bands of SOD2 and quantitative analysis results. (c) Representative bands of cleaved caspase-3 and quantitative analysis results. (d) Representative bands of Bax and quantitative analysis results. (e) Representative bands of Bcl-2 and quantitative analysis results. Values are expressed as mean ± SEM. ^*∗*^*P* < 0.05 I/R group versus Sham group; ^#^*P* < 0.05 I/R + H group versus I/R group; *n* = 6 per group.

To further confirm that amifostine might protect myocardium from I/R injury via inhibiting oxidative stress and apoptosis, we detected antioxidant and apoptosis related proteins (SOD1, SOD2, cleaved caspase 3, Bax, and Bcl2) in each group. I/R enhanced the expression of cleaved caspase 3 and Bax compared with the Sham group, whereas the expression of SOD1, SOD2, and Bcl2 was significantly decreased. Pretreatment with high dose amifostine significantly decreased the expression of cleaved caspase 3 and Bax and enhanced SOD1, SOD2, and Bcl2 expression (Figure 7).

### 3.8. Effect of Amifostine Pretreatment on SOD Activity and MDA Level

**Figure 8:**
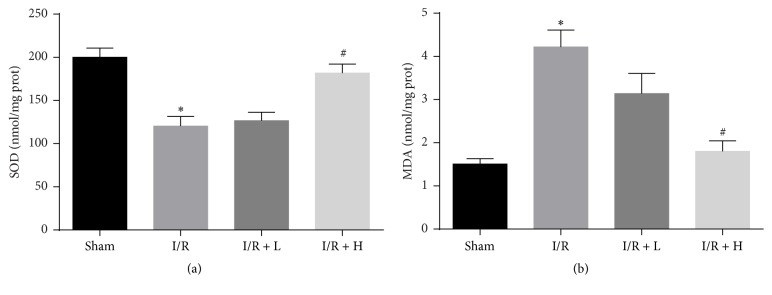
Amifostine pretreatment increases SOD activities and alleviates MDA release. (a) SOD activities. (b) MDA leaves. Values are expressed as mean ± SEM. ^*∗*^*P* < 0.05 I/R group versus Sham group; ^#^*P* < 0.05 I/R + H group versus I/R group; *n* = 6 per group.

In order to verify the assumption that amifostine alleviates myocardial induced oxidative stress, we measured SOD activities and MDA levels in the heart tissue. We found that pretreatment with amifostine significantly increased the SOD activity and reduced MDA level (Figure 8).

### 3.9. Effect of Amifostine Pretreatment on Reactive Oxygen Species (ROS) and SOD2

**Figure 9:**
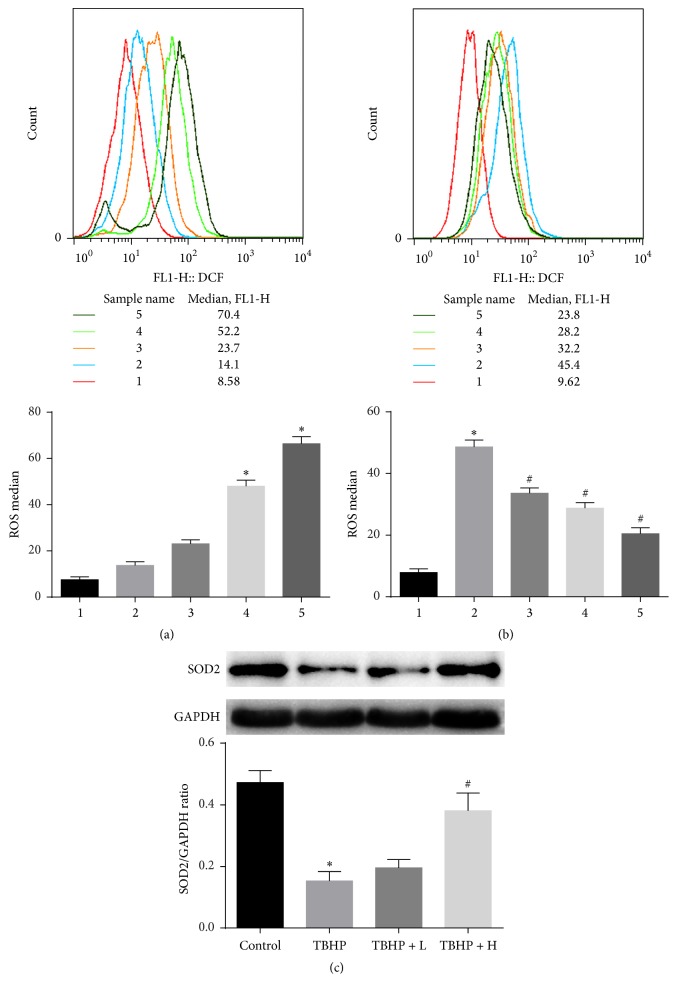
Amifostine pretreatment reduces reactive oxygen species in TBHP-induced H9c2 cells and enhances the expression of SOD2. (a) Typical images of different TBHP concentrations on reactive oxygen species in H9c2 cells. 1: control; 2: 50 *μ*M TBHP; 3: 100 *μ*M TBHP; 4: 200 *μ*M TBHP; 5: 300 *μ*M TBHP. (b) Typical images of H9c2 cells subject to amifostine pretreatment. 1: control; 2: 200 *μ*M TBHP; 3: 5 *μ*M amifostine; 4: 10 *μ*M amifostine; 5: 20 *μ*M amifostine. (c) Representative bands of cleaved SOD2 and quantitative analysis results. Values are expressed as mean ± SEM. ^*∗*^*P* < 0.05 versus control group; ^#^*P* < 0.05 versus 200 *μ*M TBHP group; *n* = 6 per group.

Generation of reactive oxygen species (ROS) increases during myocardial I/R and it plays an important role in the pathogenesis of myocardial I/R injury. The ROS level was significantly higher in H9c2 cardiomyocytes which cultured with TBHP (Figure 9(a)). As expected TBHP-induced increase in ROS production was significantly suppressed by amifostine pretreatment (Figure 9(b)). TBHP lowers the expression of SOD2 and pretreatment with high dose amifostine significantly enhanced the expression of SOD2. Taken together, these dates demonstrated that amifostine can reduce ROS.

## 4. Discussion

In this study, we found that amifostine reduced ROS level, improved cell viability, and alleviated cell apoptosis. In vivo, amifostine increased the SOD activity and reduced MDA level in I/R mice, decreased myocardial infarct size, reduced cell apoptosis and the expression of proapoptotic related proteins, and reduced DHE staining and the expression of SOD related proteins. Our results suggest that amifostine pretreatment has a protective effect against myocardial ischemia-reperfusion injury.

Myocardial I/R injury is an inherent response to the recovery blood flow after ischemia [19]. This is a complicated process including numerous mechanisms occurring in the intracellular and extracellular environments, and part of that is mediated by ROS [20]. ROS can be produced from several major sources, like cytochrome oxidase, unsaturated fatty acid oxidation, mitochondrial oxidation, and so on. And the most important ROS involved are O^2−^, hydrogen peroxide (H_2_O_2_), hypochlorous acid (HCLO), and OH^−^ [21, 22]. When the balance between the production and scavenging of ROS is perturbed an irreversible damage to cells may occur, eventually leading to cell apoptosis and necrosis [23]. Therefore, antioxidant agents have been proposed to treat myocardial ischemia reperfusion.

Amifostine is a kind of widely used normal cell protective drug, approved by the United States Food and Drug Administration (FDA). At present amifostine is mainly used in attenuating the side effects induced by radiotherapy and chemotherapy in patients with malignant tumors [24–26]. In vivo, Chronidou et al. found that amifostine could attenuate I/R injury of rabbits' spinal cord as an oxygen free radical scavenger [27]. In vitro, amifostine could protect rat heart myocytes against doxorubicin [28]. In addition, Jia et al. found that amifostine can reduce the apoptosis rate of PC12 cells induced by glutamate [29]. In another investigation, WR-1065, the active form of amifostine, can reduce apoptosis of HL-60 cells induced by etoposide [30]. Therefore, we investigated if amifostine could protect I/R-induced heart injury as an antioxidant. We found that amifostine could reduce ROS and increase SOD2 expression in TBHP-treated H9c2 cells. Mitochondria has been identified as a major source of ROS, and accumulation of ROS will cause abnormal ΔΨ*m* [31]. We found that amifostine was effective in preventing a decrease in ΔΨ*m* in TBHP-induced H9c2 cells. In vivo, amifostine could increase the SOD activity and reduce MDA level in myocardial tissue and increase the expression of SOD related proteins, DHE as a fluorescent marker of ROS, and amifostine reduces the DHE staining. Thus, amifostine may protect myocardial I/R injury via maintaining the balance of intracellular oxidants and antioxidants.

There are some limitations in our study. First, H9c2 cell used in our study is a rat cardiomyocytes derived from the heart tissue of embryonic BD1X rats, exhibiting the characteristics of a number of skeletal muscle cells. Therefore, adult murine cardiomyocytes may be more indicative of amifostine's potential use. Second, amifostine is a cytoprotective drug that can cause cytotoxic effects at high concentrations, so medications should be administered at moderate doses. Third, we used t-butyl hydroperoxide to produce an oxidative stress state for H9c2 to mimic cellular hypoxia and reoxygenation injury, which is somewhat different from real myocardial ischemia reperfusion. Fourth, in this study, we used amifostine before to induce ischemia in a preventive manner; this pretreatment approach somehow is not in line with the clinical; in fact, myocardial infarction has occurred to most of the patients before admission, so we should try to study the effect of treatment during myocardial infarct, and this is what will do in our next plan.

## 5. Conclusion

In conclusion, we demonstrated that amifostine protected myocardial cells from ischemia-reperfusion injury. Furthermore, we showed that amifostine pretreatment reduced ROS level in TBHP-treated H9c2 cells. Our finding suggests that amifostine improve myocardial I/R injury by reducing oxidative stress and subsequent cellular apoptosis.
